# Patellar Transskeletal Traction for the Treatment of Chronic Patellar Pseudoarthrosis

**DOI:** 10.1155/2019/5915701

**Published:** 2019-01-22

**Authors:** José Leonardo Rocha de Faria, Dieno Mol Souza Portella, Victor Elias Titonelli, Naasson Trindade Cavanellas, Rodrigo Pires e Albuquerque, Eduardo Branco de Sousa, João Maurício Barretto

**Affiliations:** ^1^Knee Surgery Center, National Institute of Traumatology and Orthopedics of Brazil, 500 Brazil Ave., Rio de Janeiro 20940-070, Brazil; ^2^National Institute of Traumatology and Orthopedics of Brazil, 500 Brazil Ave., Rio de Janeiro 20940-070, Brazil

## Abstract

Patellar fractures, which constitute approximately 1% of bone lesions, may lead to severe impairment of the extensor mechanism. When conservative or surgical treatment fails, the patella may develop pseudoarthrosis. Neglect or delayed treatment of this type of injury may lead to significant diastasis between the patellar fragments. There is no consensus regarding the best treatment for such cases. This study is aimed at describing a rare case of patellar pseudoarthrosis in a patient who underwent two-step surgical treatment comprising transskeletal patellar traction followed by osteosynthesis with a tension band. A 17-year-old male patient presented with a left patellar fracture that resulted from a fall from a standing height 8 years ago. He did not undergo any type of surgical treatment during that time, but the fracture was immobilized for only 2 weeks. The two-step surgical treatment with transskeletal patellar traction and patellar osteosynthesis was performed and provided satisfactory functional clinical results in this patient. This two-step surgical treatment can be performed in cases similar to ours with satisfactory results.

## 1. Introduction

Patellar pseudoarthrosis, an uncommon entity in developed countries, is not as rare in underdeveloped or developing countries such as India, Brazil, and China [1]. The incidence of pseudoarthrosis or delay in patellar consolidation ranges from 1.6% to 12.5% [2, 3]. Delayed treatment and difficulty in accessing health services because of financial and/or geographic reasons are some of the reasons for the relatively high incidence of this pathology in these aforementioned countries [1].

Neglect or delayed treatment may lead to significant diastasis among the bone fragments of pseudoarthrosis. The gap between these fragments develops more frequently in transverse fractures. In this type of injury, the constant contraction of the quadriceps performed over time causes the proximal fragment of the fracture to rise up [1, 4, 5].

The treatment of patellar pseudoarthrosis with significant diastasis between bone fragments is always challenging, and few studies on this topic have been reported in the literature. Garg et al. [1] described a surgical technique for reducing diastasis between bone fragments of pseudoarthrosis. They first performed transskeletal patellar traction in patients with a distance greater than 3 cm between the fragments, followed by osteosynthesis of the patella with a tension band, and they obtained adequate functional results.

This study is aimed at describing a rare case of chronic patellar pseudoarthrosis with 9 cm diastasis between the bone fragments in a patient who underwent two-step surgical treatment based on evidence reported by Garg et al. [1].

## 2. Case Presentation

**Figure 1:**
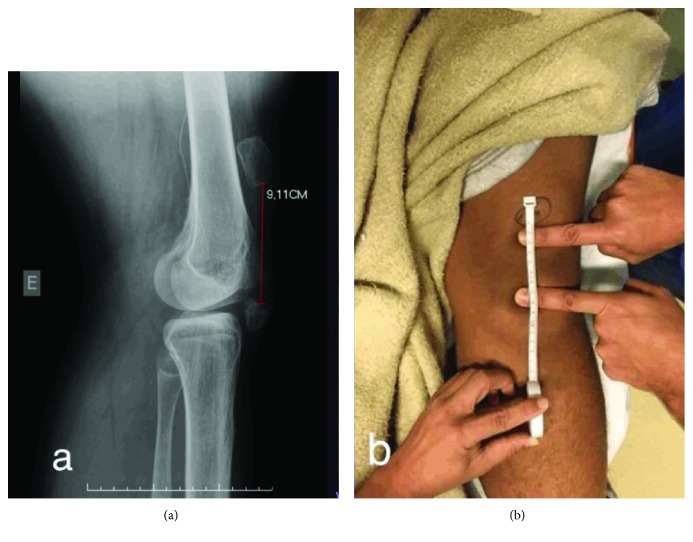
(a) Preoperative radiograph. (b) Preoperative clinical examination.

A 17-year-old male patient presented with a left patellar fracture that resulted from a fall from a standing height 8 years ago. He did not undergo any type of surgical treatment during that time, but the fracture was immobilized with crural-crustal plaster, albeit for only 2 weeks. At the present consultation, the patient presented with an active range of motion (ROM) of 70° to 120° and passive ROM of -5° to 120° (Figures 1(a) and 1(b)).

**Figure 2:**
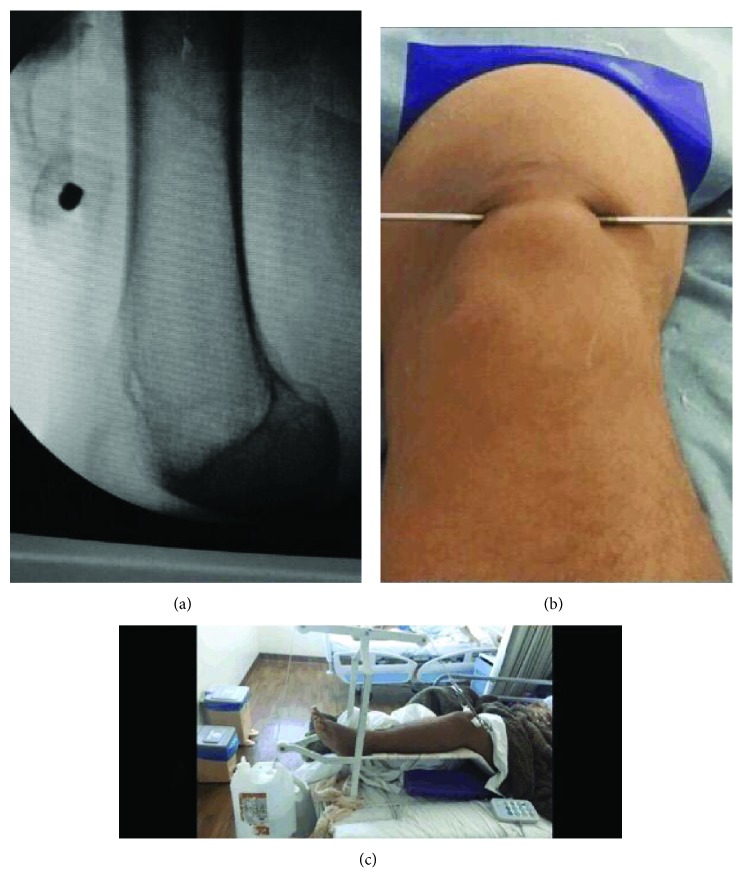
The traction pin is positioned (a, b), and traction is applied (c).

In the first phase, transskeletal patellar traction was performed, and a Steinmann pin with a 3.5 mm thick central thread (Figures 2(a)–2(c)) was inserted transversely into the proximal pole. Transskeletal patellar pin assembly is very easy to perform with the patient under sedation and local anesthesia.

**Figure 3:**
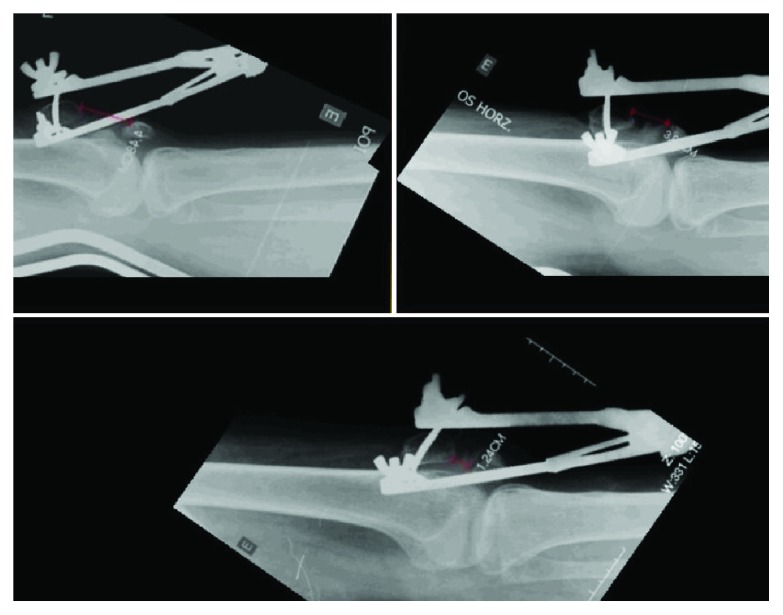
Serial radiological images.

**Figure 4:**
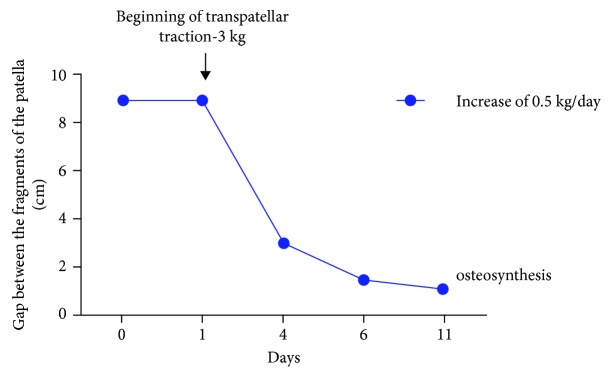
Measurements of the approximate distance between the bone fragments of the patella during transpatellar traction based on radiological images of the knee in full extension.

The traction device placed on the patella had an initial weight of 3 kg, which was increased daily by 0.5 kg. Serial radiological images were obtained to quantify the decrease in the distance between the two poles of the area of pseudoarthrosis (Figure 3). On day 11, diastasis between the fragments, which was 9 cm preoperatively, was reduced to 1.2 cm with the knee in full extension (Figure 4).

**Figure 5:**
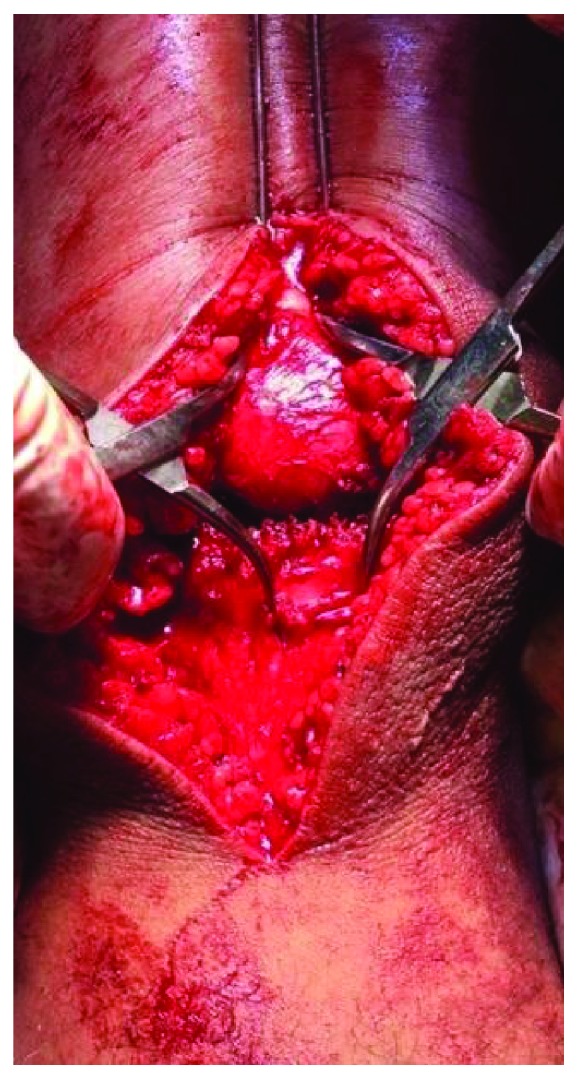
Contact between the fragments is not possible.

Then osteosynthesis was performed with a tension band. We removed the traction device and the traction pin from the proximal pole of the patella, with the patient under spinal anesthesia with femoral nerve block. We performed median longitudinal surgical access and plane dissection and identified bone fragments of the patella. We passed two 2.0 mm thick Kirschner wires, longitudinally and parallelly, into the two fragments. We attempted to reduce the fragments with two Backhaus clamps (Figure 5), but the contact between the fragments was not possible.

**Figure 6:**
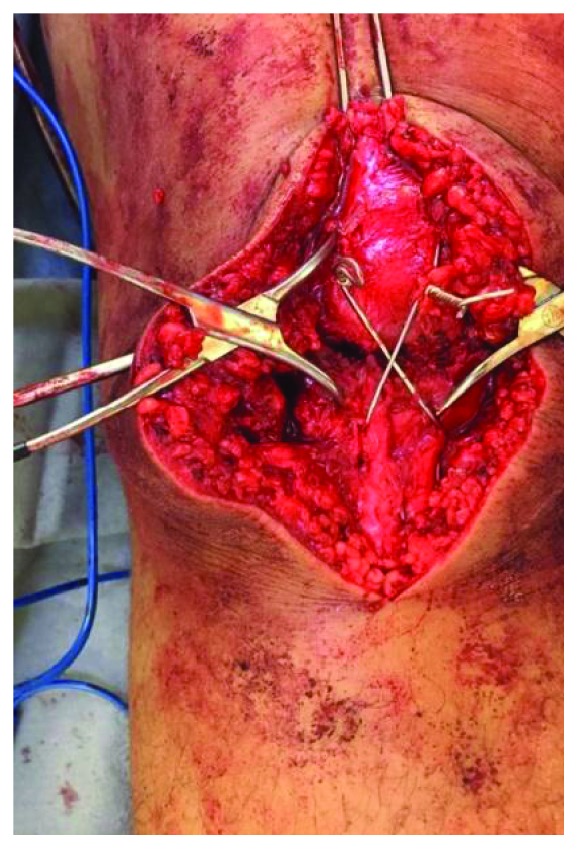
Cerclage wiring with the figure-of-eight tension band and two Backhaus clamps are used in attempt to reduce the distance between the pseudoarthrosis foci.

We performed cerclage wiring with a 1.2 mm thick cerclage wire followed by a figure-of-eight tension band. This technique considerably reduced the distance between the pseudoarthrosis foci, but the contact was insufficient (Figure 6).

**Figure 7:**
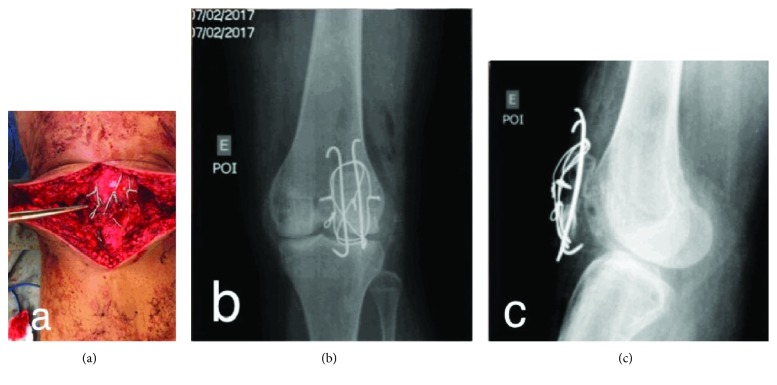
(a) Good contact between the fragments of the patella with autologous spongy graft. Radiographs in the anteroposterior (b) and lateral (c) views of the left knee in the immediate postoperative period.

Next, we proceeded with an insertion of a 1.2 mm circular cerclage bonding wire, and finally, we achieved good contact between the bone fragments of the pseudoarthrosis. After thoroughly cleaning the site with 0.9% physiological saline, we focused on the pseudoarthrosis, an autologous spongy osseous graft from the proximal tibia, and removed it through a small osseous hole made in the anteromedial tibia (Figures 7(a)–7(c)).

The patient was reassessed during the postoperative period for bone consolidation (assessed by radiography), pain (visual analog scale (VAS), 0-10), quadriceps strength (motor force rating) [6], and ROM of the limb (using a goniometer).

**Figure 8:**
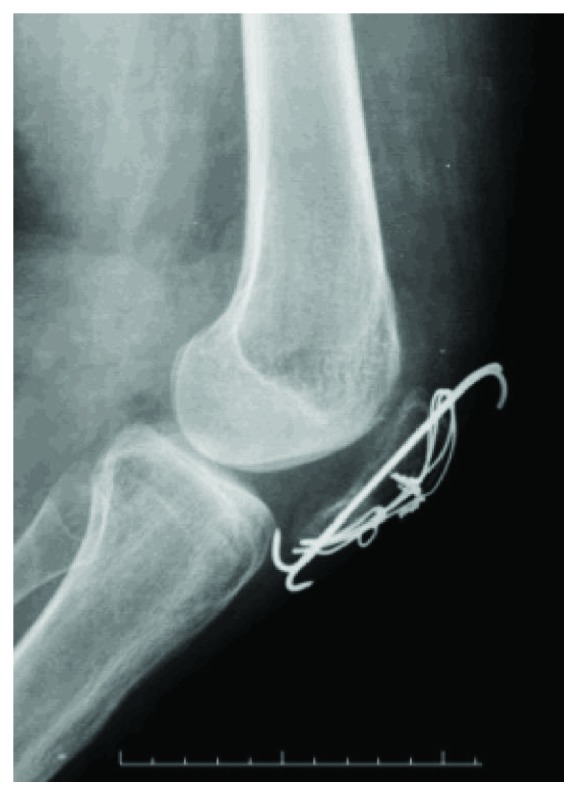
Radiograph obtained during postoperative month 3.

**Figure 9:**
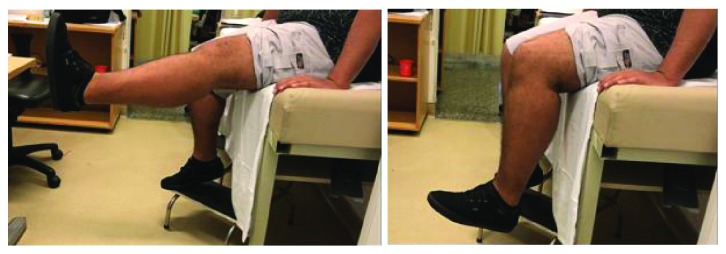
Clinical examination at 18 months postoperatively.

The patient was not immobilized and allowed full weight bearing with crutches, but knee flexion was restricted for 8 weeks. During week 8, physiotherapy focused on increasing the patient's ROM. During this period, the patient had a passive arch of 0° × 10°, grade III quadriceps strength, and a VAS score of 4, and radiography showed signs of bone consolidation. During postoperative month 3, a passive ROM of 0° × 25°, active ROM of 10° × 25°, grade IV quadriceps strength, VAS score of 4, and consolidated patella on radiography (Figure 8) were observed. During postoperative month 18, the patient had an active and passive ROM of 0° × 90° (Figure 9), grade V quadriceps strength, and VAS score of 1.

## 3. Discussion

It is challenging to reestablish suitable knee function in a case of patellar pseudoarthrosis with a significant distance between the patellar fragments. Nathan et al. [7] performed a systematic review of the management of patellar pseudoarthrosis. Most of the analyzed patients who developed pseudoarthrosis were treated with some type of immobilization. The maximum duration of pseudoarthrosis was 34 months. The maximum distance between the observed bone fragments was 8 cm. The authors did not find any study in which patellar traction was used as a treatment, and they concluded that patients with low physical demand can be treated conservatively. Individuals who require increased physical demands at work or in sports should undergo surgical treatment, and the best choice is open reduction with internal fixation and a tension band. Partial or total patellectomy can also be performed with inferior but acceptable results, according to Nathan et al. [7]. Gwinner et al. [8] conducted a review of the concepts of the treatment of patellar fractures and reached similar conclusions as Nathan et al. [7] in relation to the treatment of cases of patellar pseudoarthrosis. We believe that conservative treatment is a bad option, as it leads to poor knee function due to extension lag, which causes difficulty in activities of daily living.

Singhal et al. [9] in 2010 and Paxinos et al. [10] in 2017 published case reports in which an external fixator was used or to treat patellar pseudoarthrosis with diastasis similar to our case. Singhal et al. [9] performed external fixation of only the patella for 14 days, whereas Paxinos et al. [10] used monolateral fixation, with proximal pins in the femur, distal tibia, and intermediate patella, for 3 months. The authors of both studies reported good results with their respective techniques. The disadvantage is the possibility of an increased rate of infection at the pin sites. Hence, we preferred to use only one pin.

In 2014, Kadar et al. [11] published a retrospective cohort study with 188 patients and concluded that patients with previous stroke have a statistically significantly higher chance of developing patellar pseudoarthrosis and infection. In a meta-analysis performed in the Hospital for Special Surgery in New York on the rate of reoperation, pseudoarthrosis, and infection after patellar osteosynthesis, Dy et al. [3] concluded that age, sex, or the surgical technique has no statistically significant influence on the rates mentioned above.

In Garg et al. study [1], 35 patients with patellar pseudoarthrosis with diastasis greater than 3 cm between bone fragments were treated and divided into three groups. The first group comprised 10 patients who underwent single-stage surgical treatment, which involved V-Y quadriceps stretching and tension band osteosynthesis. The second group comprised 15 patients who underwent two-stage surgery, which involved patellar transskeletal traction (mean, 8 days) and osteosynthesis with a tension band after the space between the patellar fragments was reduced by approximately 1 cm. The third group comprised 10 patients who underwent two-stage surgery, which involved transskeletal patellar traction and partial distal pole patellectomy or total patellectomy. In all aspects evaluated, group 2 showed superior results. Thus, the authors concluded that the treatment of choice for cases of patellar pseudoarthrosis with diastasis is osteosynthesis with a tension band preceded by transskeletal traction of the patella. We achieved good results with transskeletal patellar traction and patellar osteosynthesis in our patient, and the method appears to be effective in the treatment of old neglected patellar fractures.

Two-step surgical treatment with transskeletal patellar traction and patellar osteosynthesis can be performed in cases similar to ours with satisfactory results.

## References

[1] Garg P., Satyakam K., Garg A., Sahoo S., Biswas D., Mitra S. (2012). Patellar nonunions: comparison of various surgical methods of treatment. *Indian Journal of Orthopaedics*.

[2] Klassen J. F., Trousdale R. T. (1997). Treatment of delayed and nonunion of the patella. *Journal of Orthopaedic Trauma*.

[3] Dy C. J., Little M. T. M., Berkes M. B. (2012). Meta-analysis of re-operation, nonunion, and infection after open reduction and internal fixation of patella fractures. *Journal of Trauma and Acute Care Surgery*.

[4] Elattar O., Coleman S. H., Warren R. F., Rozbruch S. R. (2016). Neglected patellar tendon rupture with massive proximal patellar migration treated with patellar transport and staged allograft reconstruction: a report of 2 cases. *Orthopaedic Journal of Sports Medicine*.

[5] Isiklar Z. U., Varner K. E., Lindsey R. W., Bocell J. R., Lintner D. M. (1996). Late reconstruction of patellar ligament ruptures using Ilizarov external fixation. *Clinical Orthopaedics and Related Research*.

[6] De Leffert R. D. (1988). Brachial plexus. *Green’s Operative Hand Surgery, Chapter 38*.

[7] Nathan S. T., Fisher B. E., Roberts C. S., Giannoudis P. V. (2011). The management of nonunion and delayed union of patella fractures: a systematic review of the literature. *International Orthopaedics*.

[8] Gwinner C., Märdian S., Schwabe P., Schaser K.-D., Krapohl B. D., Jung T. M. (2016). Current concepts review: fractures of the patella. *GMS Interdisciplinary Plastic and Reconstructive Surgery DGPW*.

[9] Singhal V., Mittal D., Lal H., Khare R. (2010). Gap non-union of patella: a treatment dilemma. *Pb Journal of Orthopaedics*.

[10] Paxinos O., Karamitros A., Douras P., Kouris N. (2018). Neglected patella nonunion successfully treated after 8 years by quadriceps distractive lengthening with a spanning unilateral external fixation system. *Knee Surgery, Sports Traumatology, Arthroscopy*.

[11] Kadar A., Sherman H., Glazer Y., Katz E., Steinberg E. L. (2015). Predictors for nonunion, reoperation and infection after surgical fixation of patellar fracture. *Journal of Orthopaedic Science*.

